# Treating Thalassemia Patients with Luspatercept: An Expert Opinion Based on Current Evidence

**DOI:** 10.3390/jcm12072584

**Published:** 2023-03-29

**Authors:** Filomena Longo, Irene Motta, Valeria Pinto, Andrea Piolatto, Paolo Ricchi, Immacolata Tartaglione, Raffaella Origa

**Affiliations:** 1 Unità Operativa di Day Hospital della Talassemia e delle Emoglobinopatie, Azienda Ospedaliero-Universitaria di Ferrara, 44124 Cona, Italy; 2 Centro Microcitemie, Azienda Ospedaliera Universitaria San Luigi Gonzaga, 10043 Orbassano, Italy; andrea.piolatto@unito.it; 3 SC Medicina Indirizzo Metabolico, Fondazione IRCCS Ca’ Granda Ospedale Maggiore Policlinico, 20122 Milano, Italy; irene.motta@unimi.it; 4 Dipartimento di Scienze Cliniche e di Comunità, Università degli Studi di Milano, 20122 Milano, Italy; 5 Centro della Microcitemia e delle Anemie Congenite, Ente Ospedaliero “Ospedali Galliera”, 16128 Genova, Italy; valeria.pinto@galliera.it; 6 Malattie Rare del Globulo Rosso, AORN Cardarelli, 80131 Napoli, Italy; paolo.ricchi@aocardarelli.it; 7 Ematologia ed Oncologia Pediatrica, AOU Policlinico Università degli Studi della Campania “Luigi Vanvitelli”, 80138 Napoli, Italy; immacolata.tartaglione@unicampania.it; 8 SC Centro Microcitemie e Anemie Rare, Ospedale Pediatrico Microcitemico ‘A. Cao’, Università di Cagliari, 09121 Cagliari, Italy; raffaella.origa@unica.it

**Keywords:** luspatercept, thalassemia, SITE, ineffective erythropoiesis, erythroid maturation agent

## Abstract

Luspatercept has recently been approved for the treatment of beta-thalassemia and its use in clinical practice has been increasing. As it is the first erythroid maturation drug available for this diagnosis, the expertise about its use is still limited. To address this point, and to promote awareness and guide the clinical use of luspatercept in beta-thalassemia, this paper was developed as a consensus by experts from the Italian Society of Thalassemia and Hemoglobinopathies (SITE). After a brief presentation of the core features of luspatercept, a comprehensive set of questions is addressed, covering relevant aspects for the practical management of this new therapeutic option.

## 1. Introduction

Luspatercept is the first and only erythroid maturation agent approved by the Food and Drug Administration (FDA) and the European Medicine Agency (EMA), acting on the latest stages of red blood cell (RBC) development process. Luspatercept is a fusion protein composed of a modified extracellular domain of activin type IIB receptor (ActRIIB), and the Fc portion of human IgG.

Although its mechanisms are not fully understood, luspatercept acts as a ligand trap, binding endogenous ligands of the TGF-β superfamily and preventing the activation of downstream effectors, including the Smad2/3 signaling pathway. This pathway is excessively activated in models of diseases characterized by ineffective erythropoiesis, i.e., myelodysplasia and beta-thalassemia. Further evidence in thalassemic mouse models showed that luspatercept reduces hemolysis and prolongs the erythrocyte lifespan [1,2,3].

The efficacy and safety of luspatercept were evaluated in the multicenter, randomized, double-blind, placebo-controlled Phase 3 BELIEVE study (ACE-536-B-THAL-001) that was performed at 65 sites in 15 countries (Australia and countries across Europe, the Middle East, North Africa, North America, and Southeast Asia) [4]. The trial enrolled adult patients (median age: 30 years) with anemia due to beta-thalassemia requiring red blood cell transfusions (6–20 units/24 weeks) without any transfusion-free period > 35 days. A total of 336 patients were randomly assigned to the luspatercept group (224 patients) or the placebo group (112 patients). The study excluded patients with hemoglobin (Hb) S/β-thalassemia or alpha-thalassemia and those with severe organ damage. Patients with a history of deep vein thrombosis, recent stroke, or had recently undergone erythropoiesis-stimulating agents, immunosuppressive therapy, or hydroxyurea therapy were also excluded [4].

Patients treated with luspatercept achieved a statistically significant decrease in transfusion requirements ≥33% compared to baseline (main endpoint), with a reduction of at least 2 units of RBC for 12 consecutive weeks, in all of the following intervals:

o From week 13 to week 24, 21.4% of patients in the luspatercept arm responded vs. 4.5% in the placebo arm (*p* < 0.001);
o From week 37 to week 48, 19.6% of patients in the luspatercept arm responded vs. 3.6% in the placebo arm (*p* < 0.001);
o In any 12-week interval, 76.3% of patients in the luspatercept arm responded vs. 34.8% in the placebo arm (*p* < 0.0001).

Moreover, luspatercept demonstrated a statistically significant reduction in transfusion requirements ≥ 33% at any interval of 24 weeks compared to placebo (45.1% vs. 2.7%, *p* < 0.0001).

When considering the more stringent endpoint of a ≥50% reduction in the transfusion requirements, the following results were achieved:

- From week 13 to week 24, 7.6% of the luspatercept arm responded vs. 1.8% of placebo (*p* = 0.03);
- From week 37 to week 48, 10.3% of the luspatercept arm responded vs. 0.9% of placebo (*p* = 0.002).

A stronger response was obtained in cases of lower baseline transfusion requirements. Relevantly, a significant percentage of the patients treated with luspatercept also achieved transfusion independence for at least 8 weeks (11.2% with luspatercept vs. 1.8% with placebo) or for at least 12 weeks (4.0% with luspatercept vs. 0% with placebo).

As for safety, the most frequent adverse reactions (reported for ≥15% of the patients each) were headache, bone pain, and arthralgia. The most commonly reported grade 3 or higher adverse drug reaction was hyperuricemia. The most severe adverse reactions included thromboembolic events such as deep vein thrombosis, ischemic stroke, portal vein thrombosis, and pulmonary embolism. Discontinuation of treatment due to an adverse reaction occurred in 2.6% of the patients treated with luspatercept [4].

Recently, luspatercept was also approved for adults with Despite its relevance, BELIEVE has some limitations: it only enrolled patients with iron input within a defined range and a limited number of transfused units. Moreover, given the different origins of the patients enrolled, they were not homogeneously transfused according to international guidelines. In addition, as is usual in clinical trials, the patients treated in BELIEVE were not representative of the whole thalassemia population, often burdened by different grades of organ dysfunction. In Italy, more than 200 patients with transfusion-dependent beta-thalassemia aged 18 years or older received at least one dose of luspatercept following EMA [5] approval and before it was marketed in the country.

Recentlynon-transfusion-dependent thalassemia (NTDT) based on the results of phase 2 BEYOND [6]. It showed that 74 of 96 patients (77.1%) in the luspatercept arm achieved a mean hemoglobin increase of 1.0 g/dL or higher from baseline over a 12-week interval during weeks 13–24 in the absence of red blood cell transfusions [6]. Given the mechanism of action of luspatercept, in this population in which transfusions do not compensate for ineffective erythropoiesis, this treatment appears particularly promising.

The present document has been developed as part of the scientific activity of the Italian Society of Thalassemia and Hemoglobinopathies (SITE), with the aim of guiding clinicians in the prescription and management of therapy in a homogeneous approach.

Up to November 2022, MEDLINE and PubMed were searched systematically for publications using the following keywords: ACE-536, luspatercept, thalassemia, ineffective erythropoiesis. Abstracts on luspatercept presented during major international scientific meetings were also taken into consideration.

## 2. Patient Selection

Patient selection is one of the major challenges for physicians, since patients who could benefit the most from new therapies are often those with a significant disease burden.

- AGE

Luspatercept is currently approved for subjects over 18 years of age.

Question 1: Is luspatercept contraindicated for specific reasons in subjects under 18?

Luspatercept is not currently indicated in the pediatric population because no data are available yet. Clinical trials are underway to evaluate the efficacy and safety in this population (NCT04143724) [7].

Question 2: Is there an upper age limit for prescribing luspatercept?

There is no upper age limit for prescribing this drug.

- GENOTYPE

Luspatercept is indicated in patients with transfusion-dependent beta-thalassemia (TDT), without genotype distinction.

Question 3: Is it necessary to analyze the patient’s genotype before starting therapy?

Sub analyses of the BELIEVE study showed that patients with a β0/β0 genotype had similar reduction in RBC transfusion burden as the overall Intention To Treat (ITT) luspatercept population [8].

*If not already available, it is suggested that the thalassemic genotype be analyzed for diagnostic confirmation and a better characterization of the treated population. However, it is not strictly required for prescription.*

Question 4: Can patients with HbE/beta-thalassemia access luspatercept therapy?

Yes, they can. The diagnosis of HbE/beta-thalassemia with a transfusion-dependent phenotype does not contraindicate the prescription of luspatercept.

Question 5: Can patients with Hb Lepore (delta–beta fusion) access luspatercept therapy?

No patients with Hb Lepore were enrolled in the phase 3 study.

*Luspatercept* should also be prescribed for patients with Hb Lepore.

Question 6: Can patients who are heterozygous for a beta-globin mutation associated with the presence of supernumerary alpha genes access luspatercept therapy?

Heterozygous patients for a beta-globin mutation associated with supernumerary alpha genes can access luspatercept therapy if they are transfusion dependent.

Question 7: Can previously non-transfusion-dependent (NTDT) patients who afterwards became TDT access luspatercept therapy?

Previously non-transfusion-dependent (NTDT) patients who afterwards developed transfusion dependence can access luspatercept therapy as long as they undergo transfusions regularly.

- IRON OVERLOAD

Question 8: Are there limits related to the values of ferritin, liver iron concentration (LIC), or myocardial iron concentration (MIC) for the administration of luspatercept?

There are no limitations related to these parameters for luspatercept prescription.

Question 9: Could luspatercept have a positive effect on iron overload?

Preliminary data show that long-term treatment with luspatercept results in a progressive reduction of serum ferritin values and a tendency to reduce the dosage of the prescribed chelation therapy [9,10,11]. Data on the effect of luspatercept on iron accumulation in the heart and liver measured by magnetic resonance (MRI) are currently insufficient to draw conclusions.

- HISTORY OF NEOPLASIA

Question 10: Is malignancy an exclusion criterion?

Patients with a history of malignancy were excluded from the clinical trials.

*Caution is advised when starting treatment with luspatercept in patients with a history of malignancy.*

- HISTORY OF THROMBOSIS

Question 11: Patients treated with luspatercept in the BELIEVE study who developed thrombosis were all splenectomized. Is splenectomy an exclusion criterion?

Splenectomy is not in itself a contraindication to luspatercept therapy.

All patients who developed thrombosis during the BELIEVE study had one or more adjunctive thrombotic risk factors, including a previous history of thrombosis.

*It is recommended that a thorough history of thrombotic risk factors is taken, and any possible correcting measure considered, particularly in splenectomized patients (i.e., to evaluate the opportunity to start acetylsalicylic acid in the function of platelet numbers as recommended by international guidelines).*

Question 12: Is thrombophilic screening required in all luspatercept candidate patients?

*Thrombophilic* screening is not recommended before starting luspatercept therapy.

Question 13: Can the splenectomized patient who has already been identified as a carrier of a mutation associated with a thrombophilic status access luspatercept therapy?

*As for the non-splenectomized patients, the decision to start luspatercept therapy or not should be evaluated on a case-by-case basis, taking the risk/benefit ratio into account.*

Question 14: If the patient had a thromboembolic event shortly before the possible start of luspatercept, is the degree of contraindication to the drug considered greater?

*Regardless of the temporal relationship with the previous thrombotic event, the panel suggests carefully evaluating subjects with previous thrombosis, considering modifiable and non-modifiable risk factors.*

- ANTICOAGULANT THERAPY

Question 15: Is anticoagulant or antiplatelet prophylaxis advisable for patients during therapy?

There is currently no evidence to support the initiation of prophylaxis with anticoagulants in patients starting treatment with luspatercept. Similarly, no scientific data support “preventive” antiplatelet treatment before starting therapy.

*Initiating luspatercept therapy is not ‘per se’ an indication to start anticoagulant or antiplatelet therapy.*

- WOMEN OF CHILDBEARING AGE

Luspatercept is contraindicated in pregnant and lactating women due to the evident teratogenic effects observed in preclinical trials. For the same reason, an effective contraceptive regimen is recommended for the duration of the therapy and 3 months after discontinuation. Women of childbearing age must be guaranteed counseling for planning a possible pregnancy.

Question 16: Is luspatercept teratogenic?

There are no data on the use of luspatercept in pregnant women. Animal studies have shown embryo–fetal toxicity.

Question 17: Do women of childbearing age have to take a pregnancy test before each administration?

It is not necessary to take a pregnancy test before each administration of luspatercept. A negative pregnancy test is required before starting therapy.

*Maintaining a particularly high focus on preventing pregnancy and promptly investigating any menstrual delay during treatment is advisable.*

Question 18: What if a woman being treated with luspatercept becomes pregnant?

If a woman treated with luspatercept becomes pregnant, the drug should be immediately discontinued. Appropriate counseling must be provided based on the existing evidence for risk assessment.

Question 19: Are there contraindications to the use of luspatercept in male subjects with a desire for offspring?

No effects of luspatercept on fertility were observed in male animals. No data are currently available on human males.

- MASSES OF EXTRAMEDULLARY ERYTHROPOIESIS

Question 20: Can luspatercept be prescribed in patients with masses of extramedullary erythropoiesis?

Extramedullary erythropoietic masses (EMH) are not an absolute contraindication to luspatercept. However, the risk/benefit ratio in each patient should be evaluated. There is currently no definitive evidence in the literature on the effect of luspatercept on extramedullary erythropoiesis. Both cases of improvement and worsening of the masses have been anecdotally reported [12].

Question 21: Is it necessary to look for possible EMH in all patients before proposing luspatercept therapy?

*The panel suggests, whenever possible, to search for any EMH using magnetic resonance imaging before starting luspatercept therapy.*

Question 22: Do patients with EMH require specific monitoring?

In light of the lack of data, there is no conclusive evidence about properly managing patients with foci of extramedullary erythropoiesis already present before starting therapy.

*Based on clinical experience, periodic instrumental monitoring with MRI and clinical evaluation in patients with masses of extramedullary erythropoiesis are suggested for the early detection of any volumetric changes.*

Question 23: Should luspatercept treatment be discontinued if EMH volume increases?

In case of a significant increase in the volume of the foci of extramedullary erythropoiesis, a dose reduction can be evaluated, as recommended by the drug fact sheet in cases of adverse reaction. The interruption of treatment may be considered in severe cases and in the presence of related symptoms. A reassessment of the transfusion protocol should also be considered.

Question 24: Can hydroxyurea therapy in patients with EMH also be continued in patients treated with luspatercept?

Hydroxyurea therapy can be taken during treatment with luspatercept.

*The panel suggests continuing the administration of hydroxyurea in cases where the efficacy in controlling erythropoiesis masses has already been verified, as the effect of luspatercept alone is not known.*

## 3. Modulation of Transfusion Therapy during Luspatercept Treatment

- PRE-TRANSFUSION HEMOGLOBIN

Before each administration of luspatercept, the Hb level should be assessed. In the case of red blood cell transfusion on the same day, the level of pre-transfusion Hb should be considered.

*The accurate recording of pre-transfusion Hb values is recommended.*

Question 25: Is there an ideal value of pre-transfusion Hb to be maintained during luspatercept therapy?

There is no fixed value of pre-transfusion Hb to be maintained during luspatercept therapy.

During pivotal studies, the value of pre-transfusion Hb recorded during the 24 weeks prior to luspatercept initiation was used as a target.

*The target for pre-transfusional Hb is the average of pre-transfusional Hb in the 24 weeks before treatment initiation if it is within the range recommended by the international guidelines.*

- TIMING OF LUSPATERCEPT ADMINISTRATION IN RELATION TO THE TRANSFUSION CYCLE

Question 26: At what point in the transfusion cycle should luspatercept be administered?

There are no data about the best timing for luspatercept administration in relation to transfusion. According to the pharmacokinetics of luspatercept, the onset of response is, on average, seven days after the administration, with a maintenance of three weeks, although with wide individual variability. Therefore, in patients on regular treatment, the response to therapy does not depend on the transfusion/luspatercept administration timing. The possibility of administering the drug on the same day of the transfusion represents a significant logistical advantage for the patient.

*There are no medical contraindications to the administration of luspatercept on the same day as transfusion.*

Question 27: In cases of administration on the same day of transfusion, is it preferable to administer luspatercept before, during, or at the end of the transfusion session?

In pivotal studies, the drug could be administered half an hour before transfusion or one hour after the end of it.

*It is believed that luspatercept can be administered approximately half an hour before transfusion so that any drug-related reactions can be identified. Post-transfusion administration, if any, should be performed at the end of the post-transfusion monitoring period. It is not recommended to administer luspatercept during transfusion because it would be challenging to differentiate the cause of a possible reaction.*

Question 28: During luspatercept therapy, is it preferable to increase the transfusion interval or to keep it unchanged by reducing the amount of blood transfused?

There is currently no evidence of differences in the effectiveness between the two modalities. In general, the reduction in transfusion sessions by increasing the interval length may be the preferred strategy from patients’ point of view. In any case, the indication to transfuse should be based on the international guidelines and rules for the appropriate use of blood.

Question 29: What should be done if, 21 days after the administration of luspatercept, Hb is higher than the target pre-transfusion Hb value?

If Hb is ≥ 11.5 g/dL without transfusions for at least 3 weeks, the dose should be postponed until Hb is ≤ 11.0 g/dL.

*In the case of Hb values being higher than the pre-transfusion Hb target, as far as is possible, it is recommended that the transfusion be postponed while abiding by the transfusion guidelines. It is also recommended that the transfusion parameters be carefully recorded.*

Question 30: What should be done if, 21 days after the administration of luspatercept, the Hb value is lower than expected?

The effect of luspatercept on blood consumption and Hb can be variable over time. Single or periodic fluctuations do not necessarily mean that the drug does not work or has stopped working in that patient.

*In these cases, it is recommended to evaluate alternative causes (immunization, hemolysis, bleeding, infection, or other conditions) as in patients not treated with luspatercept.*

## 4. Evaluation of the Response/Timing

In order to assess the response to luspatercept therapy, the comparison with the patient’s transfusion history in the 24 weeks prior to the beginning of the therapy is fundamental.

*Before starting luspatercept therapy, it is necessary to collect a detailed history of pre-transfusion Hb, number of transfused red blood cells units, transfused volume, and transfusion-free interval. The same parameters should be recorded during treatment. These data could be integrated with erythropoiesis markers.*

Question 31: Which parameter/s can be used to evaluate the response?

The parameter to assess whether the patient is a responder to luspatercept is the erythroid response, namely, the reduction in the transfusion burden in a fixed period.

*The reduction in the transfusion burden could be due to an extension of the transfusion interval or a reduction in the number of transfused units per session when the interval remains the same. If the pre-transfusion Hb is higher than the threshold value of that patient, the transfusion can be postponed.*

Question 32: Is the reduction in serum ferritin during treatment an indication of a positive response to luspatercept?

A reduction in the serum ferritin value that is not accompanied by a reduction in the transfusion burden cannot be used as an indication of a positive response to luspatercept therapy.

Question 33: Which reduction value of the transfusion burden defines the response to luspatercept?

In the phase 3 study, the erythroid response was defined as a reduction in transfusion burden ≥33% over the fixed 12-week period (between the 13th and the 24th week) plus a reduction of at least two units of red blood cells in the same 12-week interval compared to the 12 weeks prior to treatment.

*It is believed that the timing of the reduction in transfused units may differ from patient to patient. Therefore, the assessment of the response to luspatercept and the decision to continue the therapy should be evaluated on a case-by-case basis (for example, considering factors such as the presence of severe iron accumulation, immunizations, and other conditions).*

Question 34: When is it appropriate to verify the response to luspatercept?

In the phase 3 study, the median time to achieve the first response was within the first cycle: 12 or 24.5 days in patients who had a reduction ≥ 33% or ≥ 50%, respectively, during any 12-week interval. The post hoc analysis showed a faster response in patients with the non-β0/β0 genotype compared to patients with the β0/β0 genotype.

*It is believed that the response of each patient to luspatercept should be assessed by calculating the change in the number of units of red blood cells transfused in the period between the last two treatment cycles compared to the period of equal duration before the beginning of treatment. For the purpose of a more accurate evaluation, it is suggested that the variation is calculated, also considering the volumes of the units of transfused red blood cells.*

In the BELIEVE trial, a “late” response is described in patients considered non-responders to the timing of the primary endpoint, i.e., in the 13–24 week interval. Among the participants, 112 out of the 163 (68.7%) non-responders who continued treatment achieved a significant response between 25 and 48 weeks, that is, −1.85 RBC units/24 weeks vs. +0.21 RBC units/24 weeks in the placebo group [13]. Patients with a β0/β0 genotype, even if responding in a similar percentage compared to patients with other genotypes, presented a later response.

*In patients defined as non-responders according to the criteria of the BELIEVE protocol, in particular those with a β0/β0 genotype and despite the dose increase, the variability of the interpersonal and intra-personal response to luspatercept over time should be considered.*

## 5. Adverse Events

In clinical studies leading to the approval of the therapeutic use of luspatercept for beta-thalassemia, the drug was very well tolerated, both in the short-term (BELIEVE) trial and in the long-term follow-up, as shown in the subsequent analyses. The adverse events with at least 5% higher incidence in the luspatercept group than in the placebo group were bone pain, arthralgia, dizziness, hypertension, and hyperuricemia [4]. In the long-term follow-up, no new safety concerns were reported, and the adverse events of special interest (bone pain, hypertension, and thromboembolic events) were comparable with those highlighted in BELIEVE after a median duration of treatment of 103.0 weeks (range 1.7–215.0) [14,15].

- PAIN

Question 35: How frequent is bone pain?

Bone pain was reported in 19.7% of patients with beta-thalassemia treated with luspatercept (in the group treated with placebo, the incidence was 8.3%). The highest incidence was recorded in the first three months of therapy (16.6%), whereas from the fourth to the sixth month, it decreased to 3.7% [4].

Question 36: How long after the injection can the patient experience pain?

No data are available, but according to our experience, patients generally report the onset of pain a few days (2–4) after the administration of the drug.

Question 37: Does luspatercept-related bone pain represent a limitation for the patient?

In most cases, bone pain did not limit the everyday activities of the patients. In the pivotal study involving 223 patients, only 3/44 events were serious, and in only one case did the pain lead to treatment discontinuation.

Question 38: What is the most effective treatment for luspatercept-related pain?

Generally, the pain is well managed with the use of first-line analgesics (e.g., paracetamol, NSAIDs). Usually, the pain subsides spontaneously within a few days.

Question 39: Is it advisable to administer paracetamol before luspatercept to prevent the pain?

There is currently no evidence to support the use of paracetamol to prevent pain. Pain usually occurs a few days after injection, does not affect all patients, and tends to be less frequent after the first 12 weeks of therapy.

Question 40: For how long is pain present?

The available data report the presence of pain in the first weeks of therapy in particular, with a significant downward trend from week to week.

- HYPERTENSION

Question 41: How frequent is the risk of hypertension in thalassemic patients treated with luspatercept?

Hypertension, defined according to international CTCAE criteria (SBP > 140 mmHg, DBP > 90 mmHg), was reported in 8.1% of patients suffering from beta-thalassemia treated with luspatercept and in 2.8% of placebo patients. In four patients (1.8%), hypertension was grade 3 (SBP ≥ 160, DBP ≥ 100) [4]. In no case, however, was it necessary to discontinue treatment.

Question 42: If a patient develops hypertension, is it advisable to interrupt treatment?

In the clinical studies, no patients needed to discontinue treatment due to the onset of hypertension.

*If the patient develops hypertension, the panel recommends the prescription of a therapy for the management of hypertension and to consider postponing the administration of the drug in the case of an adverse event.*

- GENERAL CONSIDERATIONS, SECTION IV—ADVERSE EVENTS

Question 43: Can an already hypertensive patient be a candidate for luspatercept therapy?

In the case of a history of arterial hypertension before starting luspatercept therapy, it is recommended to optimize anti-hypertensive therapy according to the guidelines.

- HYPERURICEMIA

In the pivotal study, 3 patients out of 223 (1.3%) showed increased uric acid levels during therapy with luspatercept [4].

*It is recommended that uric acid levels be periodically monitored and current indications for the management of hyperuricemia be followed.*

- NEOPLASTIC RISK

Question 44: Is there an increased risk of cancer in those receiving luspatercept treatment?

There are currently no reports of malignancies or precancerous lesions thought to be associated with luspatercept; therefore, the available data do not support the carcinogenicity hypothesis. It should be noted that luspatercept does not fall into the category of growth factors; rather, it is a factor that promotes the *maturation* of red blood cells.

- GENERAL CONSIDERATIONS

Question 45: Are there any precautions to be observed when first administering luspatercept?

There is no evidence to support premedication prior to luspatercept administration.

*It is recommended that the patient’s state of health is assessed, monitoring vital signs and hematological parameters before each administration.*

Question 46: How long should the patient be observed for possible adverse reactions after luspatercept administration?

No serious adverse reactions were reported right after luspatercept administration. Anaphylaxis was not described. At the time of injection, some patients reported burning, pain, or erythema at the injection site; other hypersensitivity reactions were periorbital edema, angioedema, and rash (hypersensitivity recorded in 4.5% of patients); in all cases, the reactions were mild [4].

*It is prudentially suggested to keep the patient in the clinic for about 15 min after the injection.*

Question 47: If a patient experiences a side effect during therapy, should the treatment be stopped?

In the pivotal studies, the dose reduction due to an adverse event was required in 5 out of 223 patients (2.2%) and the discontinuation of treatment in 5.4% of patients. In the placebo group, the corresponding percentages were 3.7% and 0.9% [4].

The drug information of luspatercept in cases of persistent grade 3 adverse reactions indicates to proceed as follows:

- Postpone the administration of the drug until the reaction has improved and then restart treatment, according to the clinician’s judgment, at the same previous dose or at a reduced dose (for example, 1 mg/Kg if the event occurred at a dosage of 1.25 mg/Kg or 0.8 mg/Kg if the event occurred at a dosage of 1 mg/Kg).
- Discontinue treatment if the adverse event is considered unacceptable.

In cases of serious adverse reactions, refer to the clinical judgment of the prescriber whether to discontinue treatment.

*At the time of therapy proposal, it is recommended that the patient be informed of the possible expected adverse events and to discuss the treatment management, including dose reduction or delays.*

## 6. Concurrent Therapies

Question 48: Are there known drug interactions between luspatercept and other compounds?

No formal clinical studies of drug interaction have been performed. The concurrent use of iron chelating agents did not affect the pharmacokinetics of luspatercept.

Question 49: Are any therapies to be modified, discontinued, or combined when a patient starts receiving luspatercept?

No. Therapies already underway should not be modified because of luspatercept. In particular, the introduction of low molecular weight heparins, aspirin, or other antiplatelet agents to prevent thrombotic events is neither justified nor supported by scientific data unless patients need them for concurrent conditions.

Question 50: Are there any therapies that contraindicate the use of luspatercept?

The exclusion criteria of the BELIEVE pivotal clinical trial included therapy with iron chelating agents, erythropoiesis-stimulating agents, and hydroxyurea started in the 24 weeks prior to the beginning of the luspatercept therapy since they are possible confounding factors in quantifying the effect of luspatercept on anemia and iron accumulation.

To date, there are no scientific data supporting the contraindication or absence of contraindications in the association of luspatercept with chronic treatment with anticoagulants for therapeutic purposes, chronic treatment with glucocorticoids and with hydroxyurea, erythropoiesis-stimulating agents, and iron chelating agents if started within 24 weeks before treatment.

## 7. Conclusions

Luspatercept is the first erythropoiesis-stimulating agent approved for beta-thalassemia. Given the complexity of the disease, the systemic complications, and the heterogeneous response to luspatercept, a patient-tailored approach is essential. Further studies are needed to optimize patients’ selection and management to impact the history of the disease and the quality of life of patients.

## Data Availability

Not applicable.
